# Population Genetics of *Odontarrhena* (Brassicaceae) from Albania: The Effects of Anthropic Habitat Disturbance, Soil, and Altitude on a Ni-Hyperaccumulator Plant Group from a Major Serpentine Hotspot

**DOI:** 10.3390/plants9121686

**Published:** 2020-12-01

**Authors:** Andrea Coppi, Alan J. M. Baker, Isabella Bettarini, Ilaria Colzi, Guillaume Echevarria, Luigia Pazzagli, Cristina Gonnelli, Federico Selvi

**Affiliations:** 1 Department of Biology, University of Firenze, 50121 Firenze, Italy; cristina.gonnelli@unifi.it; 2 Centre for Mined Land Rehabilitation, Sustainable Minerals Institute, The University of Queensland, Brisbane QLD 4072, Australia; ajmb@unimelb.edu.au (A.J.M.B.); Guillaume.Echevarria@univ-lorraine.fr (G.E.); 3 Laboratoire Sols et Environnement, Université de Lorraine/INRA, F-54000 Vandoeuvre-lès-Nancy, France; 4 Department of Biomedical Experimental and Clinical Sciences, University of Firenze, 50121 Firenze, Italy; isa.betta@libero.it (I.B.); luigia.pazzagli@unifi.it (L.P.); 5 Department of Agriculture, Food, Environment and Forestry, Laboratories of Botany, 50121 Firenze, Italy; federico.selvi@unifi.it

**Keywords:** anthropogenic disturbance, heavy metals, Ni-hyperaccumulators, polyploids, population genetics, outlier loci, ultramafic soil

## Abstract

Albanian taxa and populations of the genus *Odontarrhena* are most promising candidates for research on metal tolerance and Ni-agromining, but their genetic structure remains unknown. We investigated phylogenetic relationships and genetic differentiation in relation to distribution and ploidy of the taxa, anthropic site disturbance, elevation, soil type, and trace metals at each population site. After performing DNA sequencing of selected accessions, we applied DNA-fingerprinting to analyze the genetic structure of 32 populations from ultramafic and non-ultramafic outcrops across Albania. Low sequence divergence resulted in poorly resolved phylograms, but supported affinity between the two diploid serpentine endemics *O. moravensis* and *O. rigida*. Analysis of molecular variance (AMOVA) revealed significant population differentiation, but no isolation by distance. Among-population variation was higher in polyploids than in diploids, in which genetic distances were lower. Genetic admixing at population and individual level occurred especially in the polyploids *O. chalcidica*, *O. decipiens*, and *O. smolikana*. Admixing increased with site disturbance. Outlier loci were higher in serpentine populations but decreased along altitude with lower drought and heat stress. Genetic variability gained by gene flow and hybridization at contact zones with “resident” species of primary ultramafic habitats promoted expansion of the tetraploid *O. chalcidica* across anthropogenic sites.

## 1. Introduction

Nickel-accumulating plants are currently attracting considerable interest for both biotechnological applications and fundamental research on the genetic bases and molecular mechanisms of metal homeostasis, evolution, and adaptation to extreme environments [1,2,3]. Due to the phylogenetic constraints involved in metal accumulation ability [4], research in this field requires the use of appropriate model systems formed by closely related accumulating and non-accumulating taxa and/or species with populations from sites with high and low levels of Ni in the soil [5,6]. In Europe, broad opportunities for research and phytoextraction technologies are offered by the most diverse groups of Ni-accumulators in the continent, i.e., the Brassicaceae genera *Bornmuellera* and *Odontarrhena* (syn. *Alyssum* sect. *Odontarrhena*; [7,8,9,10,11]. The latter genus consists of nearly 90 species ranging from the Iberian peninsula to Iran and adjacent regions [12], of which about 60 are able to accumulate Ni well above 1000 μg g^−1^ dw ([13]; Global Hyperaccumulator Database; http://hyperaccumulators.smi.uq.edu.au/collection/; [2]). Such plants are often obligate endemics of Ni-rich ultramafic outcrops (mostly “serpentine” soils) in given regions, while others grow either on or outside these outcrops and include both accumulator and non-accumulator populations [7,14]. Previous studies showed that accumulator and non-accumulator species do not form separate clades [15,16,17], and that no genetic differentiation occurs between serpentine and non-serpentine accessions of facultative species [18]. This suggested pre-adaptive capacity to accumulate Ni when growing on ultramafics, and repeated colonization events on distinct outcrops in different regions and species complexes. However, most molecular studies on Ni-accumulating plants of this genus have focused on single obligate serpentine endemics, such as *O. bertolonii* [19] or *O. lesbiaca* [20], whilst none have examined the population genetic structure of entire species complexes including obligate and facultative serpentine taxa from ultramafic and non-ultramafic soils patchily distributed in geologically variable regions. Notwithstanding, such complexes provide a unique opportunity to examine population differentiation within and among species, and to shed light on the evolutionary dynamics of edaphic specialization, ecotypic variation, and speciation.

The present investigation focuses on this topic using the whole *Odontarrhena* group from Albania as a study system. Ultramafic areas cover 11% of this rugged country and extend from 100 to 2400 m a.s.l. [21], which makes it a major center of diversity of metal-accumulating plants (and serpentine flora in general) and a most promising land for Ni-agromining [11]. In a recent study [22] we could elucidate its complicated systematics and ascertain the presence of six taxa that were already recognized by previous students of the genus and of the Albanian flora [23,24]. Most of these taxa are allopatric and restricted to undisturbed serpentine or limestone sites in separate mountain massifs, river basins or altitudinal belts, which might involve high inter-population genetic differentiation and isolation by distance. Previous studies on single Ni-accumulator *Odontarrhena* endemics from non-insular areas [18,19] and other serpentine species [25,26,27] pointed to strong genetic differentiation between populations in relation to the patchy distribution of the ultramafic outcrops. In Albania, however, such a pattern may be blurred by *O. chalcidica* and *O. decipiens*, since these polyploid species are largely sympatric and overlapping with the others over a wide altitudinal range, on both serpentine and non-serpentine soils [22]. Their wide distribution and clear preference for anthropogenic habitats suggest that historical land-use activities (i.e., agriculture, sheep farming, mining, industrialization, urbanization) may have promoted hybridization and range expansion, in line with the disturbance hypothesis of Anderson and Stebbins [28]. Moreover, recent evidence shows that species range expansion into novel habitats, including anthropic ones, is often fostered by enhanced genetic variation gained through hybridization and introgression with resident species [29]. Thus, Albanian taxa of *Odontarrhena* provide a good opportunity to examine this topic, and to investigate the levels and drivers of genetic divergence between taxa and populations from a vast ultramafic ‘archipelago’ along altitudinal and site disturbance gradients.

Using molecular markers from the nuclear and plastid genomes, we first estimated the level of DNA sequence divergence and phylogenetic relationships of all Albanian taxa, within a wide group of mainly Balkan representatives. Then, we used the more variable dominant nuclear markers Amplified Fragment Length Polymorphisms (AFLPs) to: (1) compare the levels of genetic diversity and divergence within and between species and populations, (2) assess the possible effects of site conditions such as elevation, soil type and metal concentration (Ni, Cr, Co, Ca, Mg), and (3) test the hypothesis that human disturbance could have contributed to the shaping of genetic structure in the group by promoting hybridization. AFLPs were used also to identify deviant loci potentially involved in adaptation to serpentine soil, trace metal concentration, and elevation.

Elucidating the genetic structure in this group also pointed to the potential risk of using it for agromining applications on ultramafic outcrops outside its native range and inhabited by native *Odontarrhena* species.

## 2. Results

### 2.1. DNA Sequence Divergence and Phylogeny

**Figure 1:**
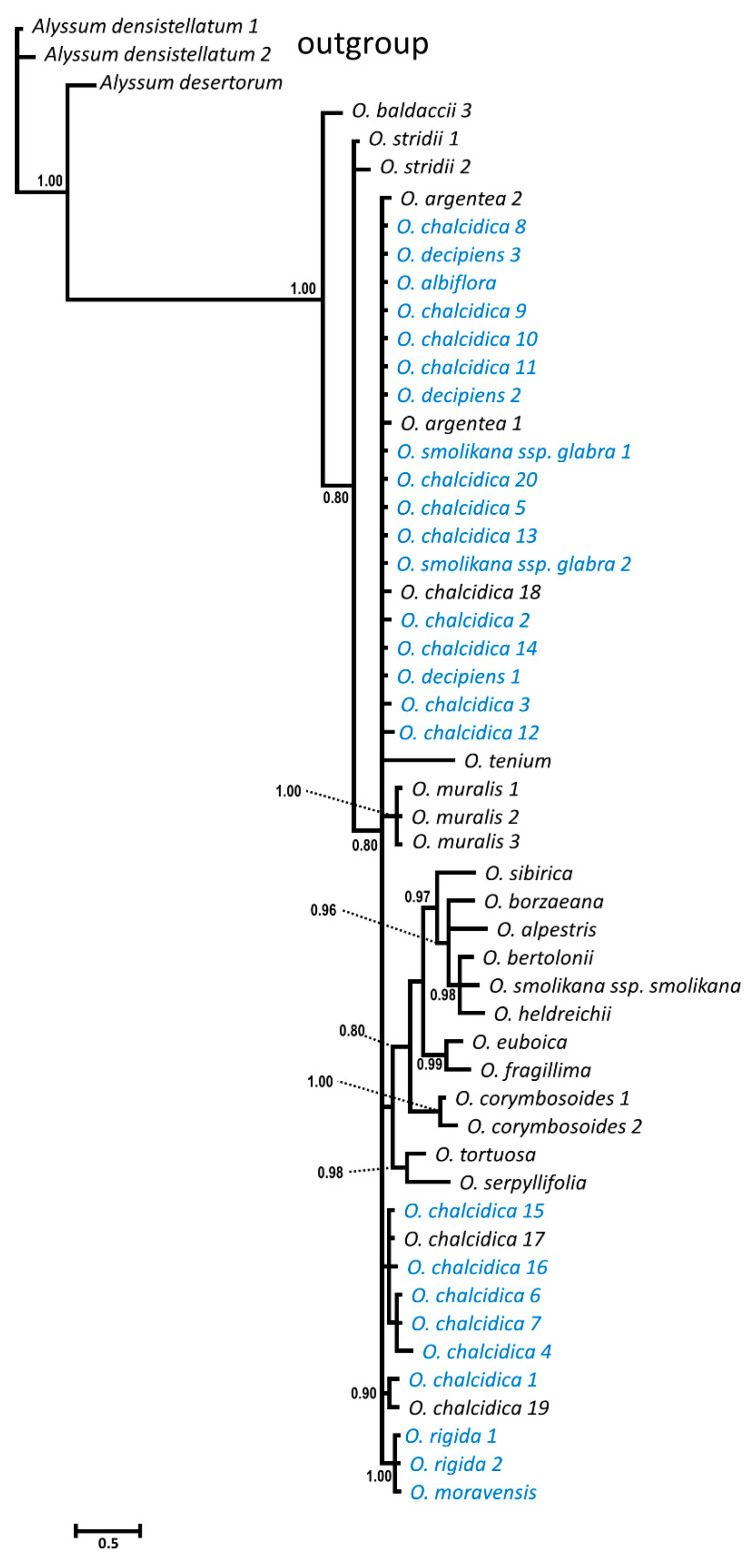
Bayesian consensus phylogram from ITS-5.8S sequences showing relationships of taxa and accessions of *Odontarrhena* from Albania (in blue). Posterior probability values > 80% are shown at the corresponding nodes.

The nuclear ribosomal DNA (rDNA) internal transcribed spacer region (ITS) alignment consisted of 629 bp (including 22 coded gaps 608-629), of which 479 conserved, 145 variable but phylogenetically non-informative and 99 (15.7%) informative; considering only the ingroup (i.e., only *Odontarrhena* accessions), only 50 positions (7.9%) were phylogenetically informative. The mean genetic distance among ingroup accessions was 0.015. The Bayesian consensus phylogram (Figure 1) retrieved *O. fallacina* from Crete as the sister to the rest of the ingroup that remained substantially unresolved.

All Albanian taxa and accessions were included in a large unresolved group. However, the two diploids *O. moravensis* and *O. rigida* formed a well-supported clade (1.00), as well as the three accessions of typical *O. muralis* from outside Albania. Small terminal clades with PP > 0.90 were formed by other Balkan species but these did not include Albanian accessions.

The chloroplast DNA trnL-trnF IGS (trnL-F) alignment was 733 bp long, (gaps in pos. 712–733). By excluding the *Alyssum* representatives (outgroup) 565 sites were conserved, 16 were variable but non-informative, and only five were (0.68%) phylogenetically informative. Mean genetic distance in the ingroup was 0.003. The IGS sequences therefore provided even less phylogenetic signal than did ITS. The resulting Bayesian tree was completely unresolved and is, therefore, not shown.

### 2.2. Genetic Structure

AFLP-fingerprinting was successfully performed on 374 individual samples and produced a total of 137 loci. The fragment length range was 41-511 bp. AMOVA showed significant differentiation between populations (FST = 0.273; *p* < 0.001) and a genetic structure dominated by within-population variation (72.44%; Table 1).

Grouping populations by species identity yielded non-significant results, whereas differences between populations within species accounted for a significant fraction of variation in the sample (26.50%; overall among-population + among-species variation = 27.56%). Among-population variation was considerably higher in tetraploid than in diploid accessions (27.0% vs. 11.2%, respectively; Table 1), and population differentiation was, therefore, stronger (FST = 0.270 vs. 0.112 in diploids).

**Figure 2:**
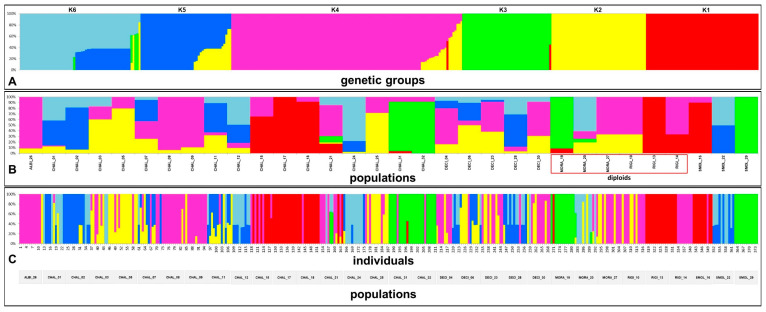
Bayesian clustering of AFLP-data from STRUCTURE, showing: (**A**) Relative proportion of each of the six genetic groups (1–6) across the 32 populations (width of the colored band); (**B**) proportion of the population membership to each of the six genetic groups; populations including more than one group (color) are admixed; diploid populations are indicated (red box); (**C**) proportion of the individual membership to the six genetic groups across the 32 populations (374 individuals); individuals including more than one group (color) are admixed.

STRUCTURE Harvester detected six genetic groups (K = 6; Supplementary Figure S1 and Table S4) and the presence of significant admixing zones (Figure 2A).

**Figure 3:**
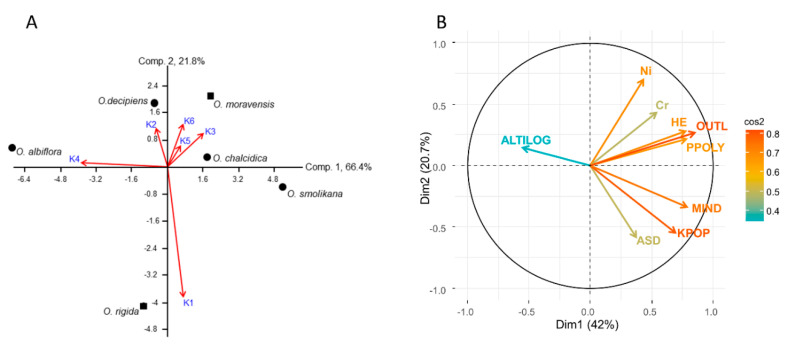
(**A**) Principal component analysis biplot showing relationships between the six genetic groups (K; numbers as in Figure 2A and the six Albanian taxa of *Odontarrhena* (dots are diploid taxa, squares are tetraploids); (**B**) Scattergram from multifactorial analysis showing the direction of variation of the number of genetic groups per population (KPOP), genetically admixed individuals (MIND), number of outlier loci (OUTL) and heterozygosity HE in relation to anthropic site disturbance (ASD), altitude (ALTILOG), and Ni and Cr soil concentration. The cos2 values, represented in the color scale barplot, show the contribution of the variables on both dimensions (Dim 1 and Dim 2).

Groups were differently represented across the 32 populations (Figure 2B) and correspondence with species identity was weak. Principal component analysis (Figure 3A) explained 88.2% of the total variation and showed that *O. rigida* and *O. albiflora* are dominated by K1 and K4, while the tetraploids *O. chalcidica*, *O. decipiens*, and *O. smolikana* include variable proportions of four to six groups.

This was the case of also *O. moravensis*, in which, however, only one of three populations (no. 20) was deviating in having four genetic groups instead of two as in pops. 19 and 27 (Figure 2B, Table 2).

Genetically admixed populations (with at least two groups both of which with ≥10% posterior probability of belonging into that genetic group) were the large majority (29 = 91%); only three populations consisted of only one group (Figure 2B, Table 2). However, diploid populations (excluding pop. 20 of *O. moravensis* mentioned above) showed a significantly lower admixing than tetraploid populations (*p* = 0.00443).

At the individual level, 75 samples (20%) included more than one genetic group (Figure 2C, Table 2); *O. albiflora* and *O. rigida* included no admixed individuals, whereas the great majority of the *O. chalcidica* accessions (14 out of 17) and all those of its hybrid *O. decipiens* included a variable proportion of admixed individuals. The two *O. moravensis* accessions no. 19 and no. 27 were formed by non-admixed individuals, while population no. 20 included five admixed individuals. When excluding the latter, the diploid profiles showed a significantly lower admixing than the tetraploids (*p* = 0.00338).

Based on multifactorial analysis (Figure 3B) the number of both genetic groups and admixed individuals were positively related to site disturbance severity. Populations from severely disturbed sites were also more admixed and consisted of more numerous genetic groups, regardless of species identity (Kruskal–Wallis test *p* = 0.0147).

Neighbor-Net analysis (Supplementary Figure S2) confirmed no clustering of individuals by species identity or population geographic origin, except for population 26 of *O. albiflora* and population 14 of *O. rigida*. Most individuals of *O. chalcidica* and *O. decipiens* were mixed with those of other species.

### 2.3. Genetic Diversity

All loci resulted as polymorphic, showing that the primer combination was effective in distinguishing all individuals of the six taxa as unique genotypes. The lowest polymorphism (32.8%) was in the population of *O. moravensis* from the type locality (no. 27), whilst the highest (87.6%) was in those of *O. decipiens* and *O. chalcidica* (Table 2). The latter taxon showed the broadest inter-population variation, due to low polymorphism in accessions 8 and 9. At the species level, polymorphism was highest in *O. smolikana* (73.5%) and lowest in *O. albiflora* (35.8%), though no significant differences existed between taxa. Diploid and tetraploid populations/taxa also did not differ significantly, but diploids had on average a lower polymorphism than tetraploids (57.9% vs. 66.8%). Similarly, polymorphism was higher in serpentine vs. non-serpentine populations (66.2% vs. 52.8%), though differences were not significant because of large variability in *O. chalcidica*. Heterozygosity was lowest in *O. albiflora* and highest in *O. smolikana*, *O. decipiens* and *O. chalcidica* (Table 2). The latter taxon again showed the largest range of infraspecific variation, due to genetically-depleted populations 8 and 9. Multifactorial analysis suggested a decrease in loci polymorphism and heterozygosity with elevation (Figure 3B), while these variables were not affected by soil type or site disturbance.

**Figure 4:**
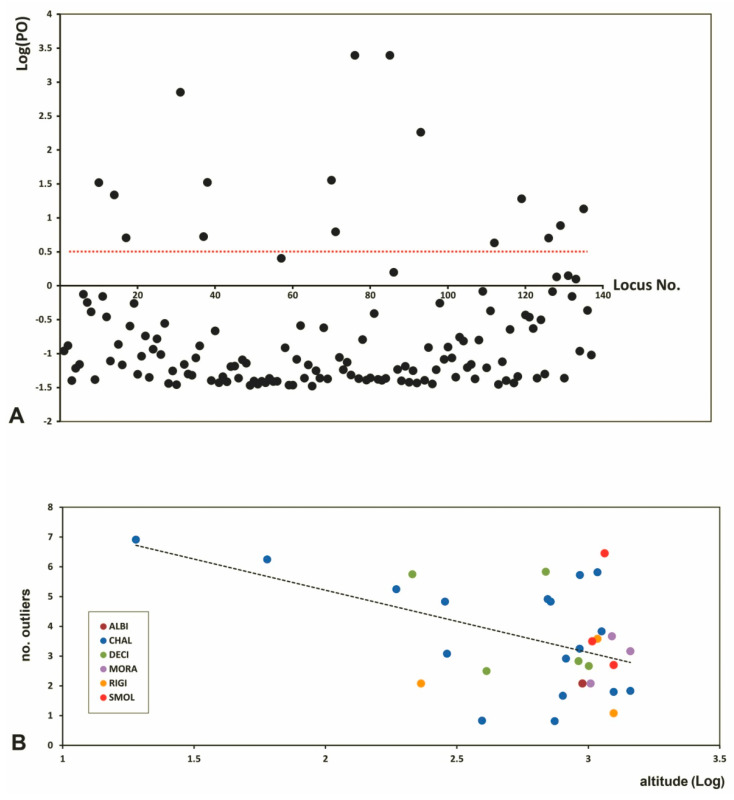
(**A**) Distribution of the 137 loci (black dots) detected by BayeScan analysis of 374 individual samples of Albanian *Odontarrhena*; dots that fall over the red threshold line [Log (posterior odds, PO) = 0.5]) are identified as outlier loci. (**B**) variation of the mean number of outliers per population with altitude (log); colours and species names abbreviations follow Figure 1 and Figure 6.

Outlier loci were 11.7% of the total (Figure 4A), their mean number per population ranging from 0.82 to 6.92 in two *O. chalcidica* accessions (Table 2).

**Figure 5:**
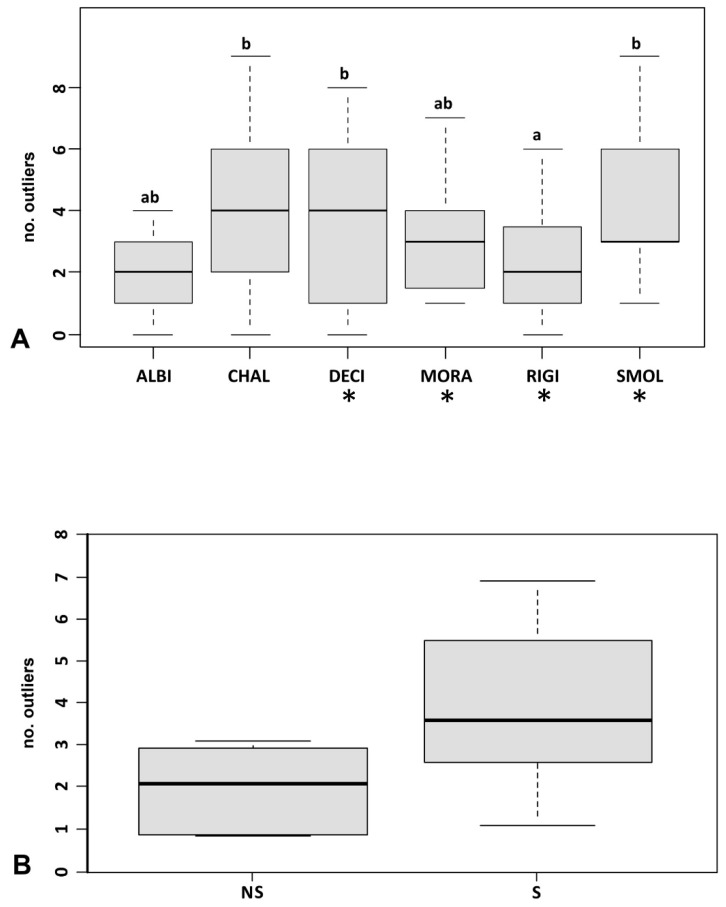
(**A**) Box-whisker plots of outlier variation across the six Albanian taxa (ALBI: *O. albiflora*; CHAL: *O. chalcidica*; DECI: *O. decipiens*; MORA: *O. moravensis*; RIGI: *O. rigida*; SMOL: *O. smolikana* subsp. *glabra*); asterisks indicate the serpentine obligate taxa, and letters indicate statistically different groups at *p* < 0.05; (**B**) variation in serpentine (S) vs. non-serpentine (NS) accessions.

At the species level, diploid *O. rigida* had significantly less outliers than tetraploids *O. chalcidica*, *O. decipiens* and *O. smolikana* (Figure 5A). However, there were no outliers exclusive to any given species, being all of them shared by at least two taxa.

Outliers were found to decrease in populations from higher elevations (*p* = 0.0096; Figure 4B), regardless of species identity, and were more represented in populations from serpentine soil (3.9 vs. 1.95 in those from other soil types; *p* = 0.0092; Figure 5B). Finally, the mean number of outliers per population tended to increase with Ni and Cr in soil (Figure 3B), though not significantly after Bonferroni correction. No relation was found with Co, Ca, Mg, ploidy level or site disturbance.

### 2.4. Genetic Distances and Gene Flow

**Figure 6:**
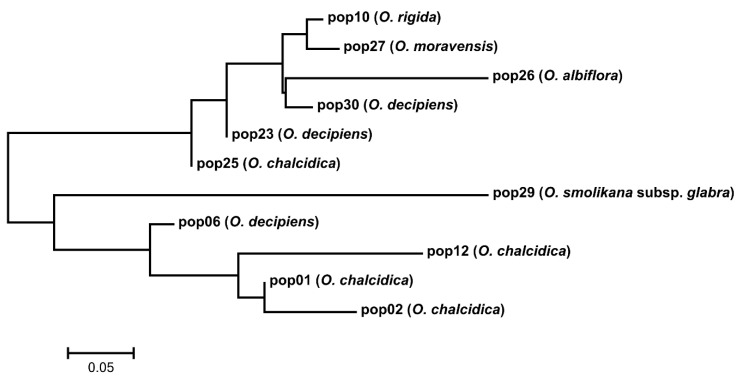
Neighbor-joining tree based on genetic distances between populations from the type localities of the taxa endemic to Albania and of those described from the same country but currently included in either *O. chalcidica* (pops. 1, 2, 12, 25) or *O. decipiens* (pops. 6, 23, 30; see Supplementary Table S1).

Slatkin’s genetic distances between populations (FST values) ranged from 0.002 to 0.956 and were not related to geographic distances (Mantel test *p* > 0.05). The NJ tree of the populations from the type localities (Figure 6) showed to main groups.

In the first one, there were both diploids (*O. rigida* and *O. moravensis* clustering together) and tetraploids (*O. albiflora*, two *O. decipiens* accessions, and one of *O. chalcidica*); the second group was formed only by tetraploids, with *O. smolikana* subsp. *glabra* distantly connected to one accession of *O. decipiens* and three of *O. chalcidica*.

**Figure 7:**
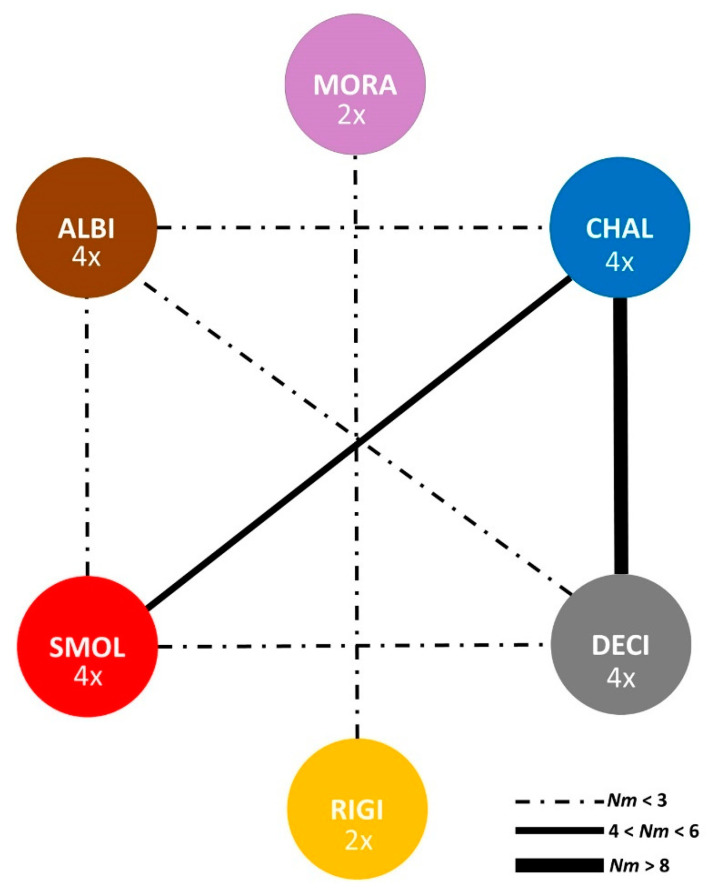
Levels of gene flow estimated as number of migrants (Nm) between the four tetraploid and two diploid Albanian taxa of *Odontarrhena*; thickness of the lines connecting the species is proportional to the estimated level of gene flow; dotted and solid lines indicate Nm values <3 (non-significant) and >3 (significant), respectively; taxon name abbreviations are as in Figure 5A.

Within single species, mean inter-population distance was highest in subalpine accessions of *O. smolikana* subsp. *glabra* (0.302) from distant massifs, whereas it was lowest in *O. decipiens* (0.224; Table 2). The estimated levels of interspecific gene flow (Nm) between tetraploids (Figure 7) showed substantial isolation of *O. albiflora* and strong exchange between especially *O. chalcidica* and *O. decipiens* (Nm = 8.9). *Odontarrhena smolikana* was more strongly connected to *O. chalcidica* (Nm = 4.2) than to their hybrid *O. decipiens* (Nm = 2.3). Gene flow between diploids was not significant (Nm = 1.98).

## 3. Discussion

### 3.1. Phylogeny

Relationships between Albanian and other taxa of *Odontarrhena* could not be satisfactorily resolved due to the low rates of variation in both ITS and trnL-F regions, in line with previous studies that suggested rapid and recent diversification as a possible cause [16,30,31]. Phylogeny provided only two relevant results. The first is that the three accessions of typical *O. muralis* from outside Albania, including one from the type locality in Romania, are clustered in a separate clade, unlike all others from this country. Most of the previous records of this important model taxon for studies on Ni-accumulation physiology and agromining applications, as well as the numerous Ni-accumulation reports from the Balkan ultramafics (i.e., [32]), should be referred to other taxa in the *O. muralis* s.l. group, mostly *O. chalcidica* or *O. decipiens*. Consequently, Ni-accumulation in *O. muralis* s. s. still requires confirmation. The second point concerns the close relationship between the two vicariant endemics *O. rigida* and *O. moravensis*, in line with their morphological synapomorphies (i.e., poorly branched cymes, glabrous silicles, unwinged seeds) and diploid chromosome complement. When looking at population genetic data, the two species showed a lower mean population heterozygosity (He) and were characterized by a lower number of genetic groups (KPop) compared with tetraploids. Remarkably, population 10 of *O. rigida* and 27 of *O. moravensis* showed an identical composition of genetic groups, accounting for their close relationship in neighbor-joining analysis and supporting their phylogenetic relationship.

### 3.2. Population Genetic Structure and Role of Anthropic Habitat Disturbance

AFLP-markers were more successful in detecting significant overall differentiation between the 32 populations analyzed, as expected from their mostly different species identity, edaphism, ploidy level, and geographic origin. Similar levels of differentiation were found in other Mediterranean endemics of *Odontarrhena*, such as Italian *O. bertolonii* [19], Aegean *O. lesbiaca* [20], and Iberian *O. serpyllifolia* [33]. As in these taxa, genetic structure of Albanian *Odontarrhena* was dominated by within-population variation, likely due to the prevalent outbreeding in the species of this genus [17]. Consistent with significant overall population differentiation, the proportion of among-population variation was not negligible (27.56%). However, this appeared lower than expected for populations that belong to six mostly allopatric taxa from the vast and geologically patchy mountainous territory of inner Albania. Moreover, geographic distance was not associated with differentiation, suggesting genetic continuity between populations along both altitudinal and latitudinal gradients, despite their different species identity and location on different soil types. Geographic distance was instead found to be positively related to population differentiation in single taxa from primary habitats, such as the obligate *O. bertolonii* and the facultative *O. serpyllifolia* mentioned above. In the latter species, distance was even more important than soil type in explaining differentiation [33], and serpentine accessions were not genetically divergent from those located on non-serpentine soils [18]. In the diploid taxa, however, genetic structure was dominated by within-population variation and overall population differentiation was lower than in the group of polyploids, in line with evidence from other plant groups [34].

A second relevant finding was that grouping of populations by species identity could not explain overall genetic variation in our sample, and that most populations consisted of two or more of these groups in variable proportions. In addition, a significant percentage of individual genetic profiles were also admixed, especially in the morphologically intermediate tetraploid hybrid *O. decipiens*. As in other plant groups [35,36] admixing of genetic groups is indicative of hybridization and introgression [37]. In our system, these mechanisms are likely responsible for the relatively high heterozygosity, polymorphism, and variability in the proportion of outlier loci in the tetraploid complex of *O. decipiens*, *O. smolikana*, and *O. chalcidica*. Introgressive hybridization especially between the former two taxa is supported by the high level of estimated gene flow between them (Nm = 8.9) and is related to their wide, mostly sympatric distribution in Northern and Central Albania, their phenotypic plasticity [22] and, possibly, the broad variation in their shoot Ni levels [38]. The role of hybridization and introgression as mechanisms driving range expansion in plants is well documented [35,39,40], and recent evidence shows that genetic variation gained through hybridization with ‘resident’ species promotes colonization of novel habitats, even in taxa that maintain their distinctness [41]. In our model system, the ‘resident’ species are the endemics from primary habitats, such as *O. smolikana*, *O. moravensis*, *O. rigida*, and *O. albiflora*, and the ‘novel’ habitat is that created by all those human land-use activities that have shaped, and still shape, large patches of the Albanian territory. Multifactorial analysis showed significantly higher levels of genetic admixing in populations and individuals of *O. chalcidica* and *O. decipiens* from anthropogenic sites, whereas narrow-ranged endemics from primary habitats such as *O. albiflora*, *O. rigida*, and *O. moravensis* (when excluding population no. 20) were much less variable in terms of genetic group composition, in line with their low phenotypic variability. As predicted by the ‘disturbance hypothesis’ [28,42], *O. chalcidica* has likely acquired adaptive genetic variability by crossing with locally sympatric ‘resident’ taxa along contact zones, widened its niche in a more generalist sense (no longer restricted to serpentine), and spread in the many non-natural sites across the country to reach an almost continuous distribution and large population size. Reduction of geographic isolation has further promoted introgressive backcrossing and the genetic swamping of the group, a process that leads to the rarefaction or extinction of pure parental genotypes though not of their alleles [43]. Anthropogenic environmental disturbance is a major driver for these processes [42], since it often involves the breakdown of ecological, geographical and behavioral barriers that isolate populations or species [43,44,45].

Further investigation should address possible hybridization events between tetraploid *O. chalcidica* and diploid *O. moravensis*, suggested by the significant admixing detected in a population (no. 20) of the latter species. This population lies at the foot of Mt. Moravë in Eastern Albania, at the contact zone between the species core range on ultramafic rocks and a plain agricultural area where *O. chalcidica* is abundant. Moreover, Cecchi et al. [22] suggested that the taxon *O. elatior* (F.K. Mey.) Španiel et al., described from this area [24], could be a hybrid between the two species, consistently with an intermediate genetic distance of pop. 25 (type locality of this taxon) between *O. moravensis* and the three other *O. chalcidica* accessions shown in the NJ tree (Figure 6). Formation of triploid bridges between polyploids and diploids have been documented in different plant groups [46,47], including *Arabidopsis lyrata* (Brassicaceae), where introgression with interploidal flow of adaptive genes can take place from diploid to tetraploid populations [48].

In revealing the complex genetic structure of *Odontarrhena* in Albania and the likely mechanisms behind it, our findings apparently provided poor molecular support to the taxa delimited based on correlation between morphology, karyology, phenology, distribution, and ecology of natural populations [22]. With the exception of *O. albiflora*, in fact, there was only partial correspondence between these taxa and the detected genetic groups, though their discrete status was already described by specialized students of this plant group and of the Albanian flora [23,24]. This result could be partly due to our more intensive sampling of polymorphic tetraploid populations from anthropogenic sites (*O. chalcidica* and *O. decipiens*) compared with less variable and geographically more restricted diploids from primary serpentine habitats (*O. rigida* and *O. moravensis*). However, the partial genetic-taxonomic discrepancy can be explained by the process of diversifying selection which allows maintenance of species (morphological) boundaries despite ongoing gene flow between them. This mechanism was recently documented in other Mediterranean species complexes with strong attitude to introgressive hybridization, such as those in the genus *Senecio* L., and is probably an underestimated speciation pathway across angiosperms [49]. This points to the need of more studies on gene flow between *Odontarrhena* populations and on the role of diversifying selection along the elevational gradient and/or levels of metal concentration in the soil or site disturbance. The persistence of morphologically distinct taxa along these gradients, as in the case of *O. chalcidica* and *O. smolikana* from low altitude to mountain slopes, could in fact be possible because this mechanism acts on only a few loci that drive adaptive speciation in spite of very low levels of genome-wide differentiation [50].

### 3.3. Genetic Diversity and Outlier Loci in Relation to Site Conditions

Although heterozygosity and polymorphism were slightly higher in serpentine populations, there were no significant deviations in the levels of genetic variability and differentiation between the accessions and taxa of *Odontarrhena* examined here. Likewise, no evidence for ecotypic differentiation was found within *O. chalcidica*, the only facultative serpentine species in our group. Reduced heterozygosity observed in some accessions of this species from severely disturbed sites (i.e., pops. 8,9) is likely due to recent founder effect events, which are known to result in the genetic impoverishment of recently established populations [51]. Other studies have pointed to a similar lack of divergence between edaphic races of facultative serpentine species in North America and Europe [26,52,53,54], suggesting that pre-adaptation to live on ultramafics is a constitutive plesiomorphic trait allowing multiple colonization events.

In our study, additional insights came from the analysis of outlier loci, which represented a considerable proportion (11.7%). Although the statistical procedure adopted here cannot be excluded to have caused false positives, we are confident that the proven robustness to this problem of AFLP data treated with Bayesian methods [55,56,57] has allowed to reduce their number to a minimum, if not to zero. While no specific outliers were associated with a given taxon in our sample, these loci were overall more numerous in populations of obligate serpentine endemics than in those from other soil types, suggesting adaptive divergence on ultramafics. Outlier proportion and expression are, in fact, directly related to adaptation in different species to environmental pressures driving divergent selection, and can indicate ecotype-level differentiation even when no divergence at neutral loci occurs [58,59,60]. The several chemical and physical anomalies of serpentine soils are known to exert a strong selection pressure on plant life [61,62,63], and recent studies have shown the genetic signatures of serpentine adaptation on different species [64,65]. Changes in outlier proportion and/or expression could, therefore, be interpreted as one of such signatures, but a recent study on *O. serpyllifolia* showed no differences between accessions from ultramafic and non-ultramafic sites across the Iberian Peninsula [33]. Moreover, our results suggest that the effects of serpentine soil on outlier frequency and expression should account for also altitude, as these loci appeared to decrease with increasing elevation of the populations investigated here. Although this finding certainly requires support from the analysis of more numerous sampling units, it is in line with evidence that elevation can affect the genetic structure of plant populations via regulation of climatic conditions [66]. Adaptive traits to tolerate drought and heat stress are typical in plants of bare serpentine outcrops of Mediterranean climatic regions [62,67,68,69], and these are particularly necessary at low altitudes because of higher temperatures and lower precipitation. In a study on genetic variation in taxa of *Senecio* on Mt. Etna, Chapman et al. [50] found an increased expression of an outlier encoding a DNAJ heat shock protein involved in response to heat stress, reflecting adaptation of low-altitude *S. chrysanthemifolius* to hotter, more arid conditions at the base of the volcano. Remarkably, Etna’s basaltic lava and serpentine soils share a very dark color, which greatly enhances heat and drought stress during spring and summer. Reduced severity of these limiting factors may therefore provide a working hypothesis to explain the decreased incidence of outliers in Albanian populations of *Odontarrhena* from higher elevations.

### 3.4. Implications for Conservation

Our findings have relevant implications for the conservation of native taxa, ecotypes, and genotypes of *Odontarrhena* across its distribution range. The conservation of metallophyte biodiversity and the identification of threats are indeed major issues that require increasing attention and ad hoc research [70]. At present, *O. muralis* s.l. populations are used in several nickel agromining experiments on ultramafic outcrops across Central and Southern Europe [11] and this may bring a new, previously unrealized threat to the genetic integrity of any local native population. When introduced in non-natural habitats, *O. muralis* s.l. is likely to be capable of crossing with adjacent congeneric populations of distinct taxa, if existing, and cause the formation of hybrids with enhanced expansion ability. We, therefore, advocate caution before introducing these populations in ultramafic areas hosting endemic *Odontarrhena* species.

## 4. Materials and Methods

### 4.1. Plant Material and Sampling Design

Species of *Odontarrhena* are small chamaephytes or long-lived caespitose herbs with branched inflorescences of numerous bright yellow flowers that are visited by a variety of pollinating insects including bees, hover flies, flies, wasps and small beetles. Allogamy is the prevailing breeding system [17], though selfing may occur, as in species of *Alyssum* [71].

Detailed information on the morphology, distribution, ecology and chromosome features in the six Albanian taxa of *Odontarrhena* are given in Cecchi et al. [22] and summarized in Table 3.

Data on Ni levels in soil and plants are also available from Bettarini et al. [38]. In summary, *O. albiflora* is a tetraploid endemic known from a single site in Eastern Albania, on calcareous cliffs at 900–1300 m a.s.l.; *O. smolikana* subsp. glabra is a tetraploid obligate serpentine endemic, occurring in rocky subalpine slopes at 1200–2200 m a.s.l.; *O. rigida* is a diploid serpentine endemic of Central Albania at 250–1200 m a.s.l., in mostly undisturbed sites; *O. moravensis* is also diploid and restricted to undisturbed serpentine sites of the E Albanian mountains, at 800–1500 m a.s.l.; *O. chalcidica* is a tetraploid species widely distributed especially in the northern, central, and eastern parts, on serpentine and other soil types (schist, flysch), at 50–1300 m a.s.l., usually in anthropogenic habitats; *O. decipiens* is a putative allotetraploid hybrid between *O. smolikana* and *O. chalcidica*, already recognized by Nyárády [23] and found at 220–1900 m a.s.l. mostly in disturbed sites of Northern and Central Albania (and Northern Greece).

**Figure 8:**
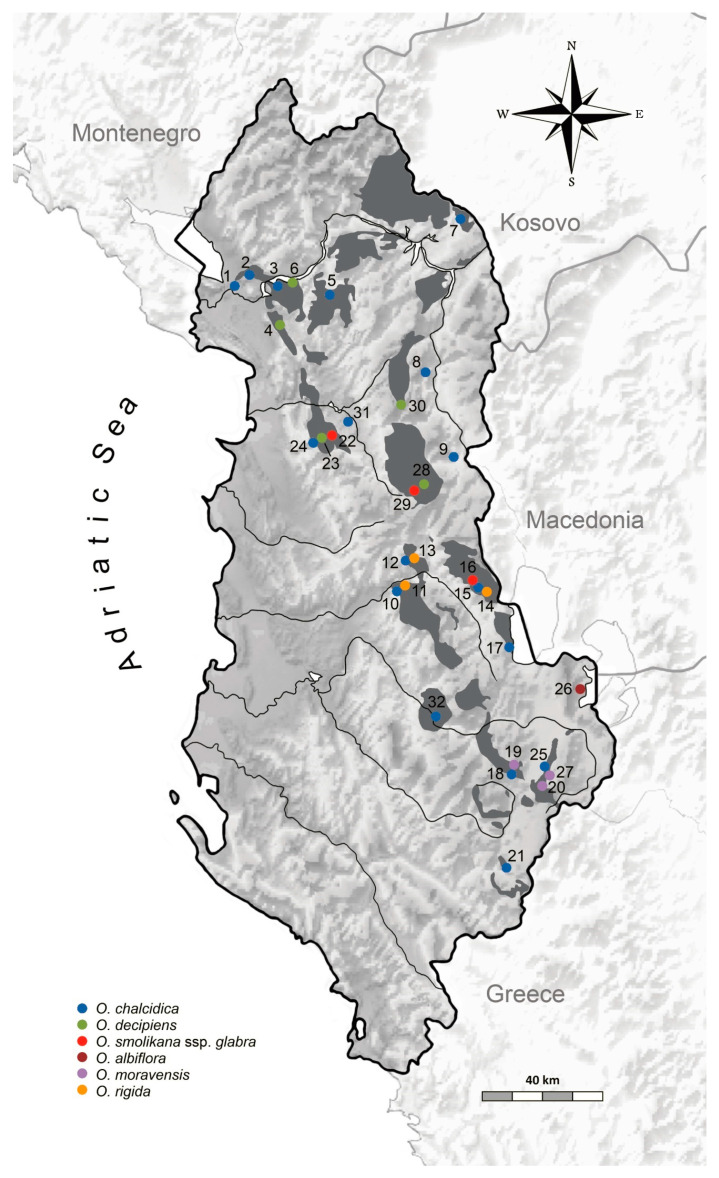
Location of the 32 sampled populations of *Odontarrhena* with species identity (colors) and distribution of the major ultramafic outcrops in Albania (dark grey spots).

During ad hoc field trips (2016, 2017, 2018) we sampled a total of 32 populations of the six taxa throughout Albania, in all major outcrops of ultramafic rocks (also indicated as “ophiolites”, especially serpentinite and harzburgite; [72]) and in sites with different soil types (Figure 8).

The number of sampling sites for each of the six taxa was unavoidably uneven due to their different frequency and range extent, from 1 (steno-endemic *O. albiflora*) to 17 (widespread *O. chalcidica*). Sampling was more intensive for *O. chalcidica* and *O. decipiens* also because these are the two taxa of the *O. muralis* group more widely used in research on metal uptake and Ni-agromining [10,11,73,74,75,76,77].

At some sites, we sampled populations of different taxa that were close to each other; hence, the distance range between populations was 0.1–230 km. For each population, leaf samples were collected randomly from 12 individuals growing at least 10 m apart on a surface of 0.5 ha, and dried in silica gel; a complete specimen was finally collected as voucher (Supplementary Table S1). Concentration of trace metals (Ni, Cr, Co, Ca, and Mg) was determined with atomic absorption spectroscopy (AAS) in soil samples from eight localities that were not previously investigated in Bettarini et al. ([38]; Supplementary Table S1; Table 4).

At each site we also recorded the severity of anthropic disturbance of the soil-vegetation system (ASD) according to an empirical ordinal scale with three levels [78]: 1 = no disturbance: natural site with primary soil; 2 = average disturbance: no signs of recent mechanical disruption of the soil but with vegetation influenced by goat or sheep grazing, or other traditional forms of anthropic use of the vegetation; 3 = heavy disturbance: non-natural site with soil texture disrupted by mostly mechanized works, such as those caused by mining, quarries, road and building construction, urbanization, intensive agriculture, and similar. Determination of ploidy level could not be performed on all sampled individuals, though karyological data were in large part available from Cecchi et al. [22] and unpublished data (Selvi, pers. obs.).

### 4.2. DNA Isolation, Amplification, Sequencing, and Phylogenetic Analysis

Genomic DNA of all 12 individual samples from the 32 populations was extracted using a modified 2xCTAB protocol [79] and quantified by a Bio-Photometer (Eppendorf; Hamburg, Germany).

For phylogenetic analysis we used the nrITS and cp trnL-F regions, both successfully applied in previous analyses of Alysseae [30,31]. Sequencing was performed on one individual sample from each of the 10 (for ITS) and nine (for trnL-F) populations representing the six Albanian taxa, including accessions from type localities (Table 3); in addition, we sequenced *O. muralis* from type locality in Romania (ITS and trnL-F) and 14 accessions of related but non-Albanian taxa for trnL-F. Additional sequences were retrieved from GenBank, bringing the total number of ingroup accessions to 50 (for ITS) and 23 (trnL-F), plus, respectively, three and two *Alyssum* outgroups (Supplementary Table S2).

ITS amplification was performed using the primers ITSp5 and ITSu4 of Cheng et al. [80], whereas the trnL-F region was amplified with the primers ‘c’ and ‘f’ of Taberlet et al. [81]. Polymerase chain reactions were performed in a total volume of 25 µL containing 2.5 µL of reaction buffer (Dynazyme II; Finnzyme, Keilaranta, Finland), 1.5 mM MgCl2, 10 pmol of each primer, 200 µM of each dNTP, 1U of Taq DNA polymerase (Dynazyme II; Finnzyme, Keilaranta, Finland) and 10 ng of template DNA. Reactions were performed in an Miniamp Thermal Cycler (Applied Biosystems, Thermo Fisher Scientific, Marsiling Industrial, Singapore). For ITS, 40 amplification cycles were run with an annealing temperature of 50 °C, annealing time of 30 s and a final extension for 45 s at 72 °C. For trnL-F, the PCR cycling conditions followed Rosati et al. [82]. Automated DNA sequencing was performed directly from the purified PCR products using BigDye Terminator v.2 chemistry and an ABI310 sequencer (PE-Applied Biosystems, Norwalk, CT, USA). The original sequences were checked for ambiguous positions based on visual inspection of the output chromatofiles and edited with BioEdit vs. 7.0 [83].

Next, the ITS and trnL-F data matrices were expanded with sequences of selected taxa retrieved from NCBI (accessions details in Supplementary Table S1). The final ITS dataset included 28 taxa and 52 accessions of which 27 were from Albania (12 original, deposited in GenBank +15 retrieved from [16]). Three *Alyssum* species were included as outgroups because of the external position of this genus in recent analyses [30,31]. The trnL-F matrix included 23 taxa/accessions of *Odontarrhena* representing all Albanian taxa, five of which were from the *O. muralis* s.l. complex; all sequences were original.

Multiple alignments were performed with MAFFT v. 7.0 (on-line version; [84]) using the Q-INS-1 strategy, which is a slow, accurate, iterative refinement method recommended for small-scale alignments [85]. Gaps were coded as separate characters according to Simmons and Ochoterena [86] using FastGap v.1.2 [87] and appended at the end of the datasets. Alignments are available from the authors upon request.

Trees were first obtained using Bayesian inference of phylogeny as implemented in MrBayes 3.1.2 [88]. Selection of the substitution models was based on the Akaike Information Criterion, using FindModel (available at: http://www.hiv.lanl.gov/content/sequence/findmodel/findmodel.html). These were GTR + Γ for ITS, with gamma-distributed rate variation across sites, and GTR + I + Γ for trnL-F.

The analyses were performed using four incrementally heated Markov chains (one cold, three heated) simultaneously started from random trees, and run for one million cycles sampling a tree every ten generations. The stationary phase was reached when the average standard deviation of split frequencies reached 0.01. Trees that preceded the stabilization of the likelihood value (the burn-in) were discarded, and the remaining trees were used to calculate a majority-rule consensus phylogram.

The trees were viewed and edited with TreeView [89], with indication of Bayesian Posterior Probabilities (PP) values for the internal tree nodes.

For both markers (ITS and trnL-F), estimates of genetic distances between the in-group accessions were calculated using the maximum complete likelihood model [90] as implemented in MEGA v.6.06 [91].

### 4.3. Population Genetic Analyses

We used amplified fragment length polymorphism (AFLPs), a still widely used tool for inferring genetic structure in plant populations because of reproducibility, robustness, and high resolving power of polymorphism [92]. This DNA-fingerprinting technique has been successfully applied to the study of plant adaptation to metal tolerance in *Arabidopsis halleri* [93], serpentine adaptation in *O. serpyllifolia* [33], and genetic structure in rare serpentine endemics of the Balkans [27]. Moreover, it proved its effectiveness also in the detection of recent introgression events in morphologically divergent taxa and in polyploid groups [94,95,96].

AFLP-fingerprinting was applied to 384 individual samples following the standard procedure with minor modifications [27].

The quality of AFLP profiles was preliminarily tested on 20 samples randomly selected from the 32 populations (ca. 5% of the total of the dataset). The preliminary AFLP protocol for quality control was performed using six primers pair combinations (Supplementary Table S3). The final primer combinations hex_EcoRI-ACG/MseI-TTA and fam_EcoRI-CTA/MseI-TTA were selected because these yielded comparable results for all of the samples, in terms of positive-PCR products and of the number and size of the peaks obtained. To limit subjectivity in the scoring of AFLP profiles [97], the AFLP profiles obtained by capillary electrophoresis for both the preliminary and final analyses were automatically scored with GeneMarker v. 1.5 (SoftGenetics LLC, PA, USA), using a cut-off value of 5% of the maximum fluorescence peak. The reproducibility of the data was assessed by replicating 20 samples that were marked as duplicated and compared with the rest of the dataset in GeneMarker during the panel editor preparation.

Following the program manual, we adopted a low peak detection threshold setting and a “stutters peak” filter to remove stutter peaks within 2.5 bp of each detected allele peak. After running the data with the size standard and the specific panel, the trace comparison report was analyzed with the duplicated samples, also checking the allele report file, and no mismatches were detected. During this step, the presence, absence, and questionable presence of alleles is shown, and in the case of complete mismatch of the peaks, the sample is automatically removed by the program to eliminate errors.

Analysis of molecular variance (AMOVA; [98]) was then performed in Arlequin v. 2.000 [99], to determine partitioning of the overall genetic variation at three levels: within-populations, among populations within species, and among (putative) taxa (i.e., all populations of putatively the same taxon grouped together). AMOVA was also performed separately for diploid and tetraploid populations.

Next, we used STRUCTURE v.2.3.3 [37] to determine the number and distribution of genetic groups and to identify admixed populations and individuals. According to Stift et al. [100], STRUCTURE is the most robust method for the analysis of genetic structure in mixed-ploidy populations analyzed with dominant markers such as AFLPs, even in groups with low population differentiation. The optimal K value (most likely number of genetic groups) was estimated using Structure Harvester [101]. The analysis was performed adopting the admixture model and 10,000 burn-ins followed by 500,000 Markov Chain Monte Carlo runs (Structure Manual). In each population, the number of genetic groups and the proportion of individual AFLP-profiles consisting of two or more genetic groups showed the level of genetic admixing as likely result of hybridization. Admixed individuals/populations were those including at least two groups, both of which with ≥10% posterior probability of belonging to that genetic group.

Principal component analysis was used to summarize and display the variation in the average proportion of each genetic group across the six groups of conspecific populations (taxa).

In addition, a Neighbor-Net analysis was performed on the matrix of uncorrected P-distances using SPLITSTREE v. 4.15.1 [102].

Genetic variation was assessed as the proportion of polymorphic loci in the dataset, and as within-population heterozygosity (He) based on Nei’s metric [103] as implemented in Arlequin v. 2.000; mean He values were averaged for conspecific populations to estimate genetic variation within species (Hs). Statistical comparisons between taxa and diploid vs. polyploid populations were made for all variables. Next, outlier loci in each individual AFLP-profile were assessed to identify genomic sites under potential selection pressure, based on differences in their frequencies between populations [104,105]. The assumption of this metric is that the locus frequencies within a population follow a multivariate β-distribution as a function of the multi-locus Fixation Index value, and the average of locus frequencies of each locus between populations [57,106,107]. Analysis in BayeScan v.2.1 [108] was carried out keeping the number of pilot runs at 20 [109], with 10,000 iterations each one [60]. Outliers are loci that fall over a threshold value set on the logarithm of posterior odds values (LogPO), determined as in Foll [108].

The mean number of outlier loci was determined for each population and the six groups of conspecific populations. After testing normality with the Shapiro–Wilk test, the six taxa were compared using ANOVA followed by a post hoc Tukey test.

Genetic distances among populations were estimated as Slatkin’s linearized pairwise FST values [110]. MEGA X [111] was then used to build a neighbor-joining tree [112] showing the distance between the typical populations from the locus classicus of the taxa described from Albania (i.e., the endemics *O. smolikana* subsp. *glabra*, *O. rigida*, *O. moravensis*, *O. albiflora*, and the “type” populations of also taxa recently included in *O. chalcidica* and *O. decipiens* as synonyms; Cecchi et al., [22]; Supplementary Table S1). Next, the genetic and geographic distances of the whole set of populations were then compared using a Mantel test as implemented in the R-package Vegan v. 2.5-6 [113]. The number of migrants per generation (Nm) was estimated separately for diploid tetraploid populations, using the Whitlock formula [114]: FST ≠ 1/(4Nm + 1).

### 4.4. Relationships between Genetic Variables and Site Conditions

The relationships between population genetic variables (polymorphic loci, He, outliers, number of genetic groups per population, and number of admixed individuals per population), site variables (altitude, soil type, metal concentration in the soil), and site disturbance were first explored using multiple factor analysis (MFA; [115]) as implemented in the package FactoMineR [116,117]. Next, we used multiple regression analysis with Bonferroni correction to test the relationships between each genetic variable above with altitude and trace metal content. Populations from low, medium, and high disturbance sites were compared for number of genetic groups using the non-parametric Kruskal–Wallis test.

## Figures and Tables

**Table 1:** Partitioning of genetic variance. AMOVA was performed at three hierarchical levels to test the differentiation between 374 individual samples from 32 populations and six species. The table shows: degrees of freedom (df), sum of squared deviations, variance component estimates, percentages of total variance contributed by each component, and the probability of obtaining a more extreme component estimate by chance alone (p). *p*-Values were estimated with 1023 permutations.

Source of Variation	df	Sum of Squares	Variance Components	Percentage of Variation	*p*-Values
Among species	5	491.962	0.23949	1.06	n.s.
Among all populations within species	26	2234.717	5.96533	26.50	<0.0001
Within all populations	342	5576.420	16.30532	72.44	<0.0001
Total	373	8302.599	22.51014		
Among diploids	1	99.78	2.26958	11.19	<0.0001
Within diploids	70	1260.250	18.00357	88.81	<0.0001
Total diploids	71	1359.958	20.27315		
Among tetraploids	25	2213.633	6.18362	26.99	<0.0001
Within tetraploids	276	4617.503	16.73008	73.01	<0.0001
Total tetraploids	301	6831.136	22.91371		

**Table 2:** Main parameters of genetic diversity in Albanian populations and taxa of *Odontarrhena*: number of genetic groups (reaching a proportion of at least 10%) in each population (KPop) and number of genetically admixed individuals (Mind), number (no. poly) and percentage (%poly) of polymorphic loci; population heterozygosity (*H*_e_), mean species heterozygosity (*H*_s_), mean number of outlier loci per population (Out) and mean genetic distance between populations within taxa (*F*_ST_).

Taxa/Pop.	KPop	Mind	No. Poly	%poly	*H*_e_	*H*_s_	Out	*F*_ST_
*O. albiflora*						0.113		—
26	2	0	49	35.766	0.113		2.08	
*O. chalcidica*						0.247		0.267
1	4	3	113	82.48	0.323	—	6.25	—
2	3	4	114	83.21	0.338	—	6.92	—
3	3	3	81	59.12	0.217	—	4.83	—
5	2	2	74	54.02	0.196	—	3.25	—
7	4	6	101	73.72	0.261	—	4.83	—
8	2	1	47	34.31	0.126	—	0.83	—
9	2	1	61	44.53	0.147	—	0.82	—
11	4	10	101	73.72	0.275	—	5.25	—
12	4	7	120	87.59	0.335	—	5.82	—
15	2	1	68	49.64	0.189	—	1.80	—
17	1	0	81	59.12	0.201	—	1.67	—
18	2	0	86	62.77	0.215	—	1.83	—
21	5	4	92	67.15	0.251	—	2.92	—
24	3	2	103	75.18	0.296	—	5.73	—
25	2	1	72	52.56	0.191	—	3.83	—
31	3	1	113	82.48	0.317	—	3.08	—
32	2	0	114	83.21	0.322	—	4.92	—
*O. decipiens*						0.231		0.224
4	4	3	81	59.12	0.185	—	2.50	—
6	4	6	96	70.07	0.252	—	5.75	—
23	4	2	78	56.93	0.199	—	2.67	—
28	4	7	120	87.59	0.348	—	5.83	—
30	3	2	69	50.37	0.171	—	2.83	—
*O. moravensis*						0.239		0.279
19	2	0	103	75.18	0.273	—	3.17	—
20	4	5	110	80.29	0.314	—	3.67	—
27	2	0	45	32.84	0.130	—	2.08	—
*O. rigida*						0.185		0.230
10	2	0	69	50.36	0.171	—	2.08	—
13	1	0	80	58.39	0.232	—	3.58	—
14	2	0	69	50.36	0.153	—	1.08	—
*O. smolikana*						0.293		0.302
16	2	0	95	69.34	0.267	—	2.70	—
22	2	4	113	82.48	0.352	—	6.45	—
29	1	0	94	68.61	0.259	—	3.50	—

**Table 3:** Taxa of genus *Odontarrhena* from Albania analyzed in the present study, with ploidy level, distribution, elevation, and habitat; based on [22].

Taxon	Ploidy Level	Distribution	Elevation (m a.s.l.)	Habitat
*O. albiflora* (F.K.Mey.) Španiel, Al-Shehbaz, D.A.German & Marhold	4x	Endemic E Albania	900–1300	Limestone cliffs
*O. chalcidica* (Janka) Španiel, Al-Shehbaz, D.A.German & Marhold	4x	Balkans; throughout Albania	50–1300	Fields, roadsides; on various soil types, more frequently on serpentine
*O. decipiens* (Nyár.) L.Cecchi & Selvi	4x	Balkans; C & N Albania	220–1900	Fields, roadsides; only on serpentine
*O. moravensis* (F.K.Mey.) L.Cecchi & Selvi	2x	Endemic E Albania	800–1500	Serpentine rocks and screes
*O. rigida* (Nyár.) L.Cecchi & Selvi	2x	Endemic C Albania	200–1250	Serpentine rocks and screes
*O. smolikana* (Nyár.) Španiel, Al-Shehbaz, D.A.German & Marhold subsp. *glabra* (Nyár.) L.Cecchi & Selvi	4x	Endemic C and N Albania	1100–2000	Serpentine rocks and screes

**Table 4:** Concentration of Ni (µg g^−1^ d.w.), Co (µg g^−1^ d.w.), Cr (µg g^−1^ d.w.), Ca (mg g^−1^ d.w.), and Mg (mg g^−1^ d.w.) in soils from the eight localities analyzed in this work. Values are the means ± standard deviation.

	Element Concentration in Soils				
Pop. Site	Ni (µg g^−1^)	Co (µg g^−1^)	Cr (µg g^−1^)	Ca (mg g^−1^)	Mg (mg g^−1^)
3 (*O. chalcidica*)	4602 ± 252	274 ± 52	2315 ± 139	1.4 ± 0.3	94.4 ± 33.1
5 (*O. chalcidica*)	3695 ± 299	240 ± 64	1951 ± 125	1.1 ± 0.4	72.2 ± 11.5
9 (*O. chalcidica*)	728 ± 13	63 ± 1	546 ± 14	0.15 ± 0.01	16.0 ± 0.6
20 (*O. moravensis*)	2451 ± 256	118 ± 4	881 ± 30	7.0 ± 0.7	95.9 ± 4.3
24 (*O. chalcidica*)	3860 ± 107	146 ± 65	550 ± 29	0.17 ± 0.03	145.3 ± 2.6
26 (*O. albiflora*)	144 ± 6	19 ± 1	155 ± 6	135.8 ± 26.9	4.9 ± 0.9
31 (*O. chalcidica*)	1098 ± 18	89 ± 17	815 ± 134	16.3 ± 2.0	109.4 ± 1.8
32 (*O. chalcidica*)	4289 ± 555	66 ± 3	678 ± 98	0.7 ± 0.1	147.9 ± 3.3

## Supplementary Materials

The following are available online at https://www.mdpi.com/2223-7747/9/12/1686/s1, Figure S1: Delta K graph showing optimal number of genetic groups, from STRUCTURE HARVESTER, Figure S2: Neighbor-Net graph of the 374 individual AFLP-profiles from SPLITSTREE, Table S1: List of accessions and taxa of Odontarrhena from Albania used for AFLP analysis, Table S2: List of taxa (in alphabetical order) and accessions included in the phylogenetic analysis, Table S3: Fluorescent labelling, primer name and primer sequence with selective extension, Table S4: Evanno table output and raw Structure output.
