# Supplementary material for: Population Genetics of Odontarrhena (Brassicaceae) from Albania: The Effects of Anthropic Habitat Disturbance, Soil, and Altitude on a Ni-Hyperaccumulator Plant Group from a Major Serpentine Hotspot
Source: Plants (Basel). 2020 Dec 1;9(12):1686. doi: 10.3390/plants9121686 (PMC7759883; doi:10.3390/plants9121686)

**Supplementary Materials**

Figure S1: Delta K graph showing optimal number of genetic groups, from STRUCTURE HARVESTER.,


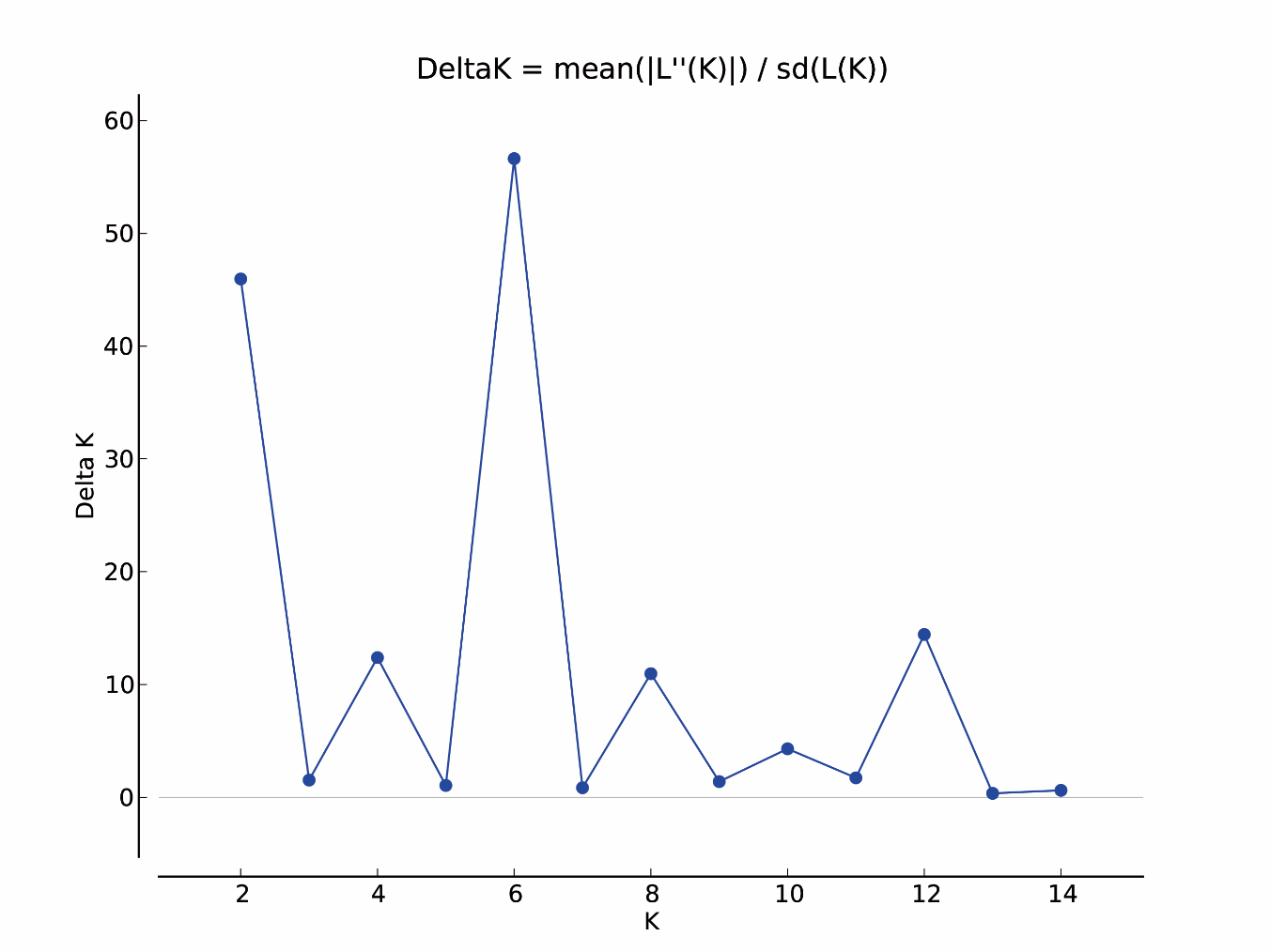

Supplement: Supplementary file 1 [file plants-09-01686-s001.zip › supplementary-revised/Supplementary Fig. S1.docx]
