# Supplementary material for: Population Genetics of Odontarrhena (Brassicaceae) from Albania: The Effects of Anthropic Habitat Disturbance, Soil, and Altitude on a Ni-Hyperaccumulator Plant Group from a Major Serpentine Hotspot
Source: Plants (Basel). 2020 Dec 1;9(12):1686. doi: 10.3390/plants9121686 (PMC7759883; doi:10.3390/plants9121686)

**Supplementary Materials**

Figure S2: Neighbor-Net graph of the 374 individual AFLP-profiles from SPLITSTREE,


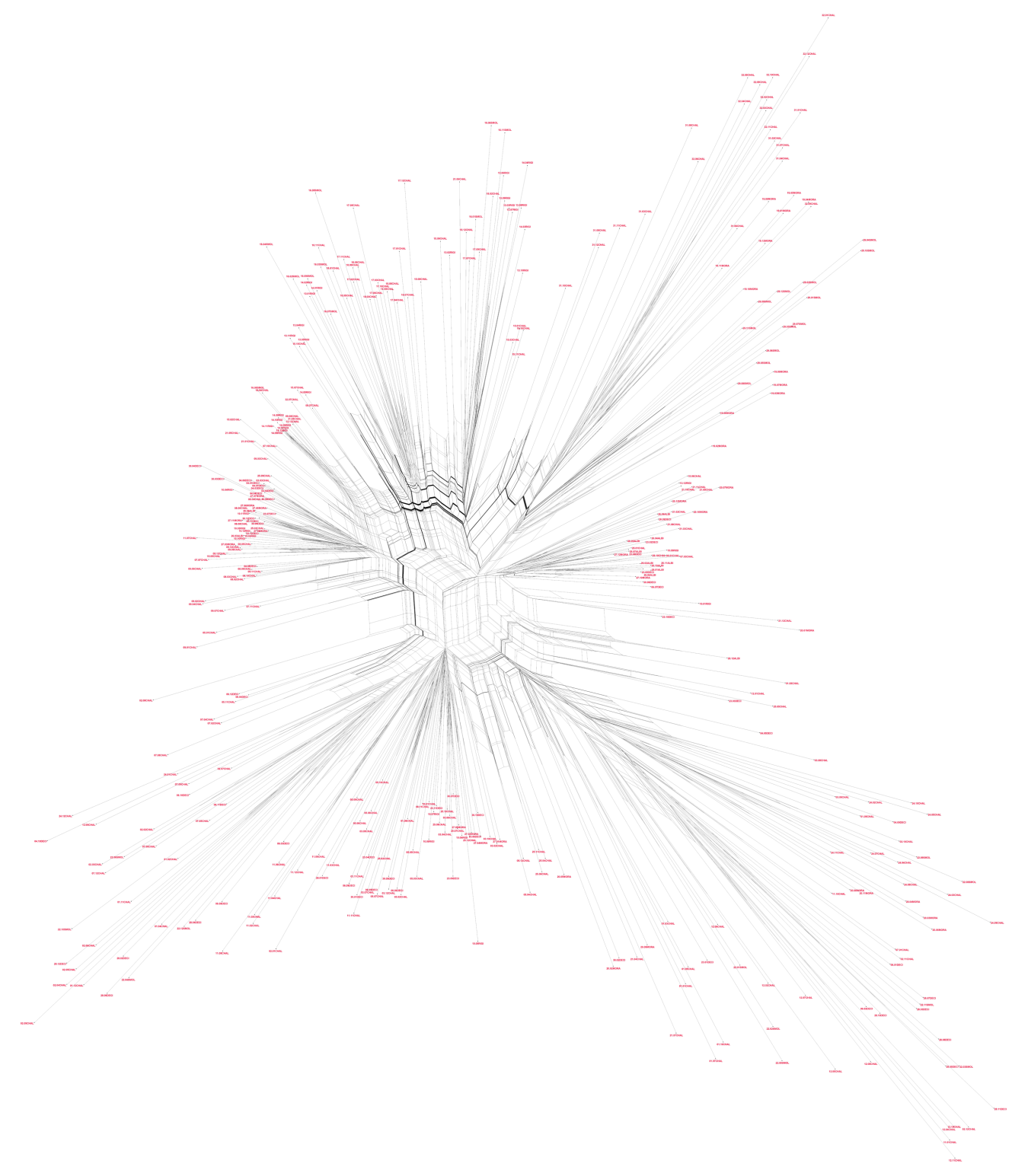

Supplement: Supplementary file 1 [file plants-09-01686-s001.zip › supplementary-revised/Supplementary Fig. S2.docx]
