# Supplementary material for: Population Genetics of Odontarrhena (Brassicaceae) from Albania: The Effects of Anthropic Habitat Disturbance, Soil, and Altitude on a Ni-Hyperaccumulator Plant Group from a Major Serpentine Hotspot
Source: Plants (Basel). 2020 Dec 1;9(12):1686. doi: 10.3390/plants9121686 (PMC7759883; doi:10.3390/plants9121686)
Supplement: Supplementary file 1 [file plants-09-01686-s001.zip › supplementary-revised/Supplementary Table 1_rev.docx]

| **Supplementary Table 1.** List of accessions and taxa of *Odontarrhena* from Albania used for AFLP analysis, with population number (Pop.), voucher specimen (all deposited in the *Herbarium Centrale Italicum* of the Florence University, FI), collection locality, geographical coordinates (Lat N, Long E), altitude, soil type and anthropic site disturbance (ASD). Authors of plant names are given in Table 1. | | | | | | | |
| --- | --- | --- | --- | --- | --- | --- | --- |
| Pop. | Taxon | Locality | Lat N Long E | Altitude m a.s.l. | Soil  Type | ASD | Voucher  (FI) |
| 1 | *O. chalcidica*^a^ | Shkodër, Renc | 42°03'46.09'' 19°32'40.24'' | 61 | Ultramafic | average | FI050421 |
| 2 | *O. chalcidica*^b^ | Shkodër | 42°03'08.60'' 19°32'01.03'' | 18 | Ultramafic | high | FI050420 |
| 3* | *O. chalcidica* | Vau-Dejës | 42°0'26.964'' 19°41'1.86'' | 285 | Ultramafic | average | FI050418 |
| 4 | *O. decipiens* | Pukë, Gjegian | 41°56’40.79'' 20°00’44.00” | 317 | Ultramafic | high | FI050443 |
| 5* | *O. chalcidica* | Pukë, Fushë-Arrëz | 42°2'8.37" 19°55'44.85" | 940 | Ultramafic | high | FI050445 |
| 6 | *O. decipiens*^c^ | Tropojë, Fierzë^1,2^ | 42°16'05.92'' 20°01'34.54'' | 214 | Ultramafic | average | FI050444 |
| 7 | *O. chalcidica* | Krumë-Kukës | 42°07'07.28'' 20°20'50.03'' | 720 | Ultramafic | high | FI050422 |
| 8 | *O. chalcidica* | Peshkopi, Muhurr | 41°43'04.08'' 20°19'21.25'' | 396 | Schist | high | FI050426 |
| 9* | *O. chalcidica* | Shupenze, Librazhd | 41°29'52.62'' 20°26'1.284'' | 740 | Schist | high | FI050425 |
| **10** | ***O. rigida*** | Elbasan, Shushice^1,2^ | 41°05'54.20'' 20°09'01.58'' | 231 | Ultramafic | absent | FI050434 |
| 11 | *O. chalcidica* | Elbasan, Shushice | 41°05'54.28'' 20°08'46.13'' | 186 | Ultramafic | high | FI050423 |
| 12 | *O. chalcidica*^d^ | Librazhd, Pishkash^1,2^ | 41°05'45.06'' 20°31'10.45'' | 1081 | Ultramafic | average | FI050424 |
| 13 | *O. rigida* | Librazhd, Pishkash | 41°05'45.06'' 20°31'10.45'' | 1081 | Ultramafic | average | FI050435 |
| 14 | *O. rigida* | Prrënjas, Mt. Shebenik | 41°09'14.08'' 20°32'18.60'' | 1248 | Ultramafic | average | FI050436 |
| 15 | *O. chalcidica* | Prrënjas, Mt. Shebenik | 41°09'14.08'' 20°32'18.60'' | 1248 | Ultramafic | average | FI050428 |
| 16 | *O. smolikana*  subsp. *glabra* | Prrënjas, Mt. Shebenik^1,2^ | 41°09'14.08'' 20°32'18.60'' | 1248 | Ultramafic | average | FI050433 |
| 17 | *O. chalcidica* | Pogradeč | 40°54'49.86'' 20°38'15.97'' | 793 | Ultramafic | high | FI050416 |
| 18 | *O. chalcidica* | Korçë, Voskopoje | 40°36'01.08'' 20°35'40.27'' | 1428 | Ultramafic | average | FI050415 |
| 19 | *O. moravensis* | Korçë, Voskopoje^1,2^ | 40°36'01.08'' 20°35'40.27'' | 1428 | Ultramafic | absent | FI050827 |
| 20* | *O. moravensis* | Korçë, Boboshticë | 40°32'41.136'' 20°47'9.024'' | 1200 | Ultramafic | absent | FI050438 |
| 21 | *O. chalcidica* | Erseke, Barmash | 40°16'22.09'' 20°36'30.60'' | 822 | Schist | average | FI050417 |
| 22 | *O. smolikana*  subsp. *glabra* | Kruje, Qafë Shtamës^1,2^ | 41°31'31.94'' 19°54'07.49'' | 1155 | Ultramafic | absent | FI050430 |
| 23 | *O. decipiens*^e^ | Kruje, Qafë Shtamës^1^ | 41°31'11.42'' 19°52'51.85'' | 1004 | Ultramafic | high | FI050442 |
| 24* | *O. chalcidica* | Kruje, Qafë Shtamës | 41°31'3'' 19°52'49.62'' | 985 | Ultramafic | high | FI050419 |
| 25 | *O. chalcidica*^f^ | Korçë, Drenovë^1,2^ | 40°34’22.43'' 20°48’23.45'' | 1121 | Ultramafic | high | FI050838 |
| **26*** | ***O. albiflora*** | Korçë, Mali Thatë^1,2^ | 40°45'26.06" 20°49'59.29" | 950 | Limestone | absent | FI050840 |
| **27** | ***O. moravensis*** | Korçë, Mt. Moravë | 40°34’43.99'' 20°47’46.56'' | 1023 | Ultramafic | absent | FI050828 |
| 28 | *O. decipiens* | Fushë Bulqizë | 41°31’02.26'' 20°15’59.20'' | 692 | Ultramafic | high | FI050834 |
| **29** | ***O. smolikana***  subsp. ***glabra*** | Bulqizë, Krastë | 41°27’33.86'' 20°10'32.65'' | 1035 | Ultramafic | absent | FI050831 |
| 30 | *O. decipiens*^g^ | Mat, Qafë Murrë^1,2^ | 41°38’03.40'' 20°07’45.90'' | 915 | Ultramafic | average | FI050829 |
| 31* | *O. chalcidica* | Burrel | 41°36.915' 20°00.170' | 260 | Schist | high | FI050842 |
| 32* | *O. chalcidica* | Upper Devoll valley | 40°43.337' 20°32.653' | 680 | Ultramafic | average | FI052169 |

The asterisk * indicate populations and sites analyzed in this work for soil content of Cr, Ni and Co; values for the other sites/populations were from Bettarini et al. (2019). Populations from type localities of Albanian endemic taxa are given in bold; superscript letters indicate populations from type localites of taxa included as synonyms of either *O. chalcidica* or *O. decipiens* after Cecchi et al. (2018): ^a^ *A. bertolonii* subsp. *scutarinum* Nyár.; ^b^ *A. jancheni* Nyár.; ^c^ *A. balkanicum* Nyár., ^d^ *A. markgrafii* O.E.Schultz, ^e^ *A. balkanicum* Nyár., ^f^ *A. elatius* F.K.Mey., ^g^ *A. balkanicum var. depressum* Nyár.

^1^ population included in the ITS phylogenetic analysis; ^2^ population included in the *trn*L-F phylogenetic analysis.
