# Supplementary material for: Population Genetics of Odontarrhena (Brassicaceae) from Albania: The Effects of Anthropic Habitat Disturbance, Soil, and Altitude on a Ni-Hyperaccumulator Plant Group from a Major Serpentine Hotspot
Source: Plants (Basel). 2020 Dec 1;9(12):1686. doi: 10.3390/plants9121686 (PMC7759883; doi:10.3390/plants9121686)
Supplement: Supplementary file 1 [file plants-09-01686-s001.zip › supplementary-revised/Supplementary Table 2.docx]

**Supplementary Table 2**. List of taxa (in alphabetical order) and accessions included in the phylogenetic analysis, with origin, voucher (herbarium acronyms are according to Index Herbariorum) and GenBank accession numbers (ITS, *trn*L-F). Original sequences are marked with an asterisk. Plant nomenclature follows Španiel *et al.* (2015). Population number of the Albanian accessions used for the population genetic analysis are given as in Table 1.

| Taxon | Origin and Voucher | ITS | *trn*L-F |
| --- | --- | --- | --- |
|  |  |  |  |
| ***Alyssum desertorum*** Stapf | Albania, Korçë, Bitincka; *Hasko* 05.07; FI | GQ284881 | — |
| ***Alyssum densistellatum*** T.R. Dudley | 1. Greece, Sterea Ellas, Evia; *Cecchi & Selvi* 08.17; FI | GQ284879 | — |
|  | 2. Greece, Sterea Ellas, Mt. Kallidhromon; *Cecchi & Selvi* 08.14; FI | GQ284880 | — |
| ***Alyssum klimesii*** [Al-Shehbaz](http://www.ipni.org/ipni/idPlantNameSearch.do?id=20008471-1&back_page=%2Fipni%2FeditAdvPlantNameSearch.do%3Ffind_infragenus%3D%26find_isAPNIRecord%3Dtrue%26find_geoUnit%3D%26find_includePublicationAuthors%3Dtrue%26find_addedSince%3D%26find_family%3D%26find_genus%3DAlyssum%2B%26find_sortByFamily%3Dtrue%26find_isGCIRecord%3Dtrue%26find_infrafamily%3D%26find_rankToReturn%3Dall%26find_publicationTitle%3D%26find_authorAbbrev%3D%26find_infraspecies%3D%26find_includeBasionymAuthors%3Dtrue%26find_modifiedSince%3D%26find_isIKRecord%3Dtrue%26find_species%3Dklimesii%26output_format%3Dnormal) | India: Ladak, Rupshu, Tso Moriri; *Klimes*; ALTB 2070 | — | FN677736 |
| ***Alyssum lenense*** Adams | Kazakhstan:Tarbagatai; *Smirnov* *et al.* 2001; ALTB B008 | — | FN677633 |
| ***Odontarhena albiflora*** (F.K. Mey.) Španiel, Al-Shehbaz, D.A. German & Marhold | Albania, Korçë, Mali Thatë; *Cecchi et al.* FI 050840 | MK775289* | MK779748* |
| ***O. alpestris*** (L.) Ledeb. | France, Hautes-Alpes, Col du Lautaret; *Bruneau* 1474; FI | AY237957 | — |
| ***O. argentea*** (All.) Ledeb. | 1. Italy, Piedmont, Vallanta; *Selvi* 08.31; FI | GQ284855 | — |
|  | 2. Italy, Piedmont, Molette; *Siniscalco* s.n.; TO | GQ284854 | — |
| ***O. baldaccii*** (Nyár.) Španiel | Greece, Crete, Gonies; *Cecchi & Selvi* 2018; FI FI055544 | MK775288 | — |
| ***O. bertolonii*** (Desv.) Jord. & Fourr. | Italia, Tuscany, Mt. Ferrato; *Cecchi & Selvi* 08.35; FI | GQ284859 | — |
| ***O. borzaeana*** (Nyár.) D.A. German | Ucraina; *Vakarenko & Mosyakin* s.n.; MO | EF514603 | — |
| ***O. chalcidica*** (Janka) Španiel, Al-Shehbaz, D.A. German & Marhold | 1. Albania, Shkodër; *Cecchi et al.* 06.13; FI 050420 (type loc. of *A. bertolonii* subsp. *scutarinum* Nyár.) – Pop. 1 | GQ284867 | — |
|  | 2. Albania, Elbasan, Librazhd; *Hasko* 06.19; FI | GQ284864 | — |
|  | 3. Albania, Elbasan, Librazhd; *Cecchi et al.* 07.21; FI | GQ284863 | — |
|  | 4. Albania, Kukës, Mt. Paštrik; *Cecchi et al.* 06.17; FI | GQ284868 | — |
|  | 5. Albania, Elbasan, Librazhd; *Hasko* 3306; FI | GQ284866 | — |
|  | 6. Albania, Elbasan, Perrënjas; *Hasko* 06.20; FI | GQ284871 | — |
|  | 7. Albania, Elbasan, Perrënjas; *Cecchi* *et al.* 07.22 | GQ284869 | — |
|  | 8. Albania, Elbasan, Perrënjas; *Hasko* 3334; FI | GQ284870 | — |
|  | 9. Albania, Korçë, Bitincka; *Hasko* 04.01; FI | GQ284861 | — |
|  | 10. Albania, Korçë, Pogradeč; *Hasko* 06.21; FI | GQ284875 | — |
|  | 11. Albania, Korçë, Pogradeč; *Cecchi et al.* 07.23; FI | GQ284872 | — |
|  | 12. Albania, Korçë, Pogradeč; *Hasko* 04.02; FI | GQ284873 | — |
|  | 13. Albania, Korçë, Pogradeč; *Hasko* 05.10; FI | GQ284874 | — |
|  | 14. Albania, Librazhd, Pishkash*, Cecchi et al.* FI050424 (type loc. of *Alyssum markgrafii* O.E. Schultz) – Pop. 12 | MK775290* | MK779749* |
|  | 15. Albania, Lezhë, Rubik; *Hasko* 3355; FI | GQ284862 | — |
|  | 16. Albania, Berat; *Hasko* 07.24; FI | GQ284860 | — |
|  | 17. Greece, Macedonia, Mt. Pangeon; Univ. Copenhagen Excurs. 226; C | GQ284886 | — |
|  | 18. Greece, Epirus, Metsovo; *Cecchi & Selvi* 08.04; FI | GQ284877 | MK779750* |
|  | 19. Bulgaria, Blagoevgrad, Mt. Belasica; *Pavlova et al.*; SOF 104630 | GQ284885 | — |
|  | 20. Albania, Drenovë; *Cecchi et al.* FI050838 (type loc. of *Alyssum elatius* F.K.Mey.) – Pop. 25 | MK775291* | MK779751* |
| ***O. corymbosoides*** (Formánek) Španiel, Al-Shehbaz, D.A. German & Marhold | 1. Greece, W Macedonia, Mt. Vourinos; *Cecchi & Selvi* 08.25; FI | GQ284878 | MK779752* |
|  | 2. Republic of Macedonia, Prilep; *Cernoch* 27287 | GQ284892 | — |
| ***O. decipiens*** (Nyár.) L. Cecchi & Selvi | 1. Albania, Fierzë; *Cecchi et al.* FI050444 (type loc. of *A. balkanicum* Nyár.) – Pop. 6 | MK775292* | MK779753* |
|  | 2. Albania, Shtamës; *Cecchi et al.* FI050442 - Pop. 23 | MK775293* | — |
|  | 3. Albania, Qafë Murrë; *Cecchi et al.*  FI050829 – Pop. 30 | MK775294* | MK779754* |
| ***O. euboea*** (Halácsy) Španiel, Al-Shehbaz, D.A. German & Marhold | Greece, Sterea Ellas, Evia; *Cecchi & Selvi* 08.20; FI | GQ284882 | MK779755* |
| ***O. fragillima*** (Bald.) Španiel, Al-Shehbaz, D.A. German & Marhold | Greece, Crete, Lefka Ori; *Bergmeier & Matthäs* 3254; C | GQ284883 | MK779756* |
|  |  |  |  |
| ***O. heldreichii*** (Hausskn.) Španiel, Al-Shehbaz, D.A. German & Marhold | Greece, Western Macedonia, Mt. Vourinos; *Cecchi & Selvi* 08.26; FI | GQ284884 | MK779759* |
| ***O. moravensis*** (F.K. Mey.) L. Cecchi & Selvi | Albania, Voskopoje; *Cecchi et al.* FI050440 - Pop.19 | MK775295* | MK779760* |
| ***O. muralis*** (Waldst. et Kit.) Endl. | 1. Romania, Deva (type locality); *Selvi* FI052171 | MK775296* | MK779761* |
|  | 2. Canada, Alberta (cultivated), *Campbell* 330 (DAO) | EF514614 | — |
|  | 3. Italy, Piedmont, (naturalized) Casteldelfino; *Selvi* 08.32; FI | GQ284887 | — |
|  | Bulgaria, *sine loc., sine coll.* (sub. *A. murale var. pichleri*); FI | — | MK779762* |
| ***O. nebrodensis*** (Tineo) L.Cecchi & Selvi | Italy, Sicily, Piano della Battaglia; *Brullo* s.d. CAT | — | MK779763* |
| ***O. orbelica*** *(*Ančev & Uzunov) Španiel, Al-Shehbaz, D.A. German & Marhold | Bulgaria, Blagoevgrad, Dunino Kuche peak; *Uzunov & Ančev*; SOM 155156 | — | MK779764* |
| ***O. rigida*** (Nyár.) L.Cecchi & Selvi | 1.Albania, Elbasan, Mt. Shpat, *Cecchi* *et al.* FI050434 - Pop. 10 | MK775297* | MK779765* |
|  | 2. Albania, Elbasan, Librazhd; *Hasko* 3303; FI | GQ284865 | — |
| ***O. serpyllifolia*** (Desf.) Jord. & Fourr. | Portugal, *Auriault* 13088; MO | EF514623 | — |
| ***O. sibirica*** (Willd). Španiel, Al-Shehbaz, D.A. German & Marhold | Greece, Sterea Ellas, Evia; *Cecchi & Selvi* 08.21; FI | GQ284890 | MK779766* |
| ***O. smolikana*** (Nyár.) Španiel, Al-Shehbaz, D.A. German & Marhold  subsp. ***smolikana*** | Greece, Epirus, Mt. Smolikas; *Cecchi & Selvi* 08.28; FI | GQ284891 | MK779767* |
| ***O. smolikana*** subsp. ***glabra*** (Nyár.) L. Cecchi & Selvi | 1. Albania, Perrënjas Mt. Shebenik; *Cecchi et al.* FI050433 – Pop 16 | MK775298* | MK779768* |
|  | 2. Albania, Kruje, Shtamës; *Cecchi et al.* FI050431 – Pop. 22 | MK775299* | MK779769* |
| ***O. stridii*** L.Cecchi, Španiel & Selvi | 1. Greece, Sterea Ellas, Kedros; *Cecchi & Selvi* 08.08; FI | GQ284856 | MK779757* |
|  | 2. Greece, Sterea Ellas, Mt. Kallidhromon; *Cecchi & Selvi* 08.12; FI | GQ284857 | MK779758* |
| ***O. tenium*** Halácsy | Greece, Cyclades, Tinos; *Baker* s.n. | AY237926 | — |
| ***O. tortuosa*** (Waldst. & Kit. ex Willd.) C.A.Mey. | Turkmenistan; *Kurbanov* 391; MO | EF514625 | — |
| ***O. troodi*** (Boiss.) Španiel, Al-Shehbaz, D.A. German & Marhold | Cyprus, Mts. Troodos, Mt. Khionistra; *Brullo et al.* s.n.; CAT | — | MK779770* |
