# Supplementary material for: Population Genetics of Odontarrhena (Brassicaceae) from Albania: The Effects of Anthropic Habitat Disturbance, Soil, and Altitude on a Ni-Hyperaccumulator Plant Group from a Major Serpentine Hotspot
Source: Plants (Basel). 2020 Dec 1;9(12):1686. doi: 10.3390/plants9121686 (PMC7759883; doi:10.3390/plants9121686)
Supplement: Supplementary file 1 [file plants-09-01686-s001.zip › supplementary-revised/Supplementary Table 3.DOCX]

| **Supplementary Table 3**. Fluorescent labelling, primer name and primer sequence with selective extension (in brackets) | | | | | |  |  |  |
| --- | --- | --- | --- | --- | --- | --- | --- | --- |
| Fluorescent end-labelling | Primer name | Primer sequence | | | | |  |  |
|  | PMseI_TTA | GATGAGTCCTGAGTAA(TTA) |  |  |  |  |  |  |
|  | PMseI_GAT | GATGAGTCCTGAGTAA(GAT) | | | | |  |  |
| 5’ hexachloro-fluorescein- phosphoramidite | hex_pEcoRI_ACG | GACTGCGTACCAATTC(ACG) | | | | |  |  |
| 5’ 6-fluorescein amidite | fam_pEcoRI_TAC | GACTGCGTACCAATTC(TAC) | | | | |  |  |
| 5’ 6-fluorescein amidite | fam_pEcoRI_CTA | GACTGCGTACCAATTC(CTA) | | | | |  |  |
