# Supplementary material for: Population Genetics of Odontarrhena (Brassicaceae) from Albania: The Effects of Anthropic Habitat Disturbance, Soil, and Altitude on a Ni-Hyperaccumulator Plant Group from a Major Serpentine Hotspot
Source: Plants (Basel). 2020 Dec 1;9(12):1686. doi: 10.3390/plants9121686 (PMC7759883; doi:10.3390/plants9121686)
Supplement: Supplementary file 1 [file plants-09-01686-s001.zip › supplementary-revised/Supplementary Table 4.DOCX]

**Supplementary Table 4.** Evanno table output and raw Structure output.

| **K** | **Reps** | **Mean LnP(K)** | **Stdev LnP(K)** | **Ln'(K)** | **\|Ln''(K)\|** | **Delta K** |
| --- | --- | --- | --- | --- | --- | --- |
| 1 | 3 | -38575.666667 | 16.427213 | — | — | — |
| 2 | 3 | -35086.700000 | 62.645271 | 3488.966667 | 2878.666667 | 45.951859 |
| 3 | 3 | -34476.400000 | 400.869043 | 610.300000 | 610.633333 | 1.523274 |
| 4 | 3 | -33255.466667 | 307.086150 | 1220.933333 | 3802.100000 | 12.381216 |
| 5 | 3 | -35836.633333 | 6867.812410 | -2581.166667 | 7185.166667 | 1.046209 |
| 6 | 3 | -31232.633333 | 81.437481 | 4604.000000 | 4611.000000 | 56.620121 |
| 7 | 3 | -31239.633333 | 682.543481 | -7.000000 | 574.800000 | 0.842144 |
| 8 | 3 | -30671.833333 | 32.262414 | 567.800000 | 353.466667 | 10.955990 |
| 9 | 3 | -30457.500000 | 419.289482 | 214.333333 | 581.833333 | 1.387665 |
| 10 | 3 | -30825.000000 | 616.310320 | -367.500000 | 2648.766667 | 4.297781 |
| 11 | 3 | -33841.266667 | 3746.866286 | -3016.266667 | 6450.233333 | 1.721501 |
| 12 | 3 | -30407.300000 | 337.011884 | 3433.966667 | 4862.900000 | 14.429461 |
| 13 | 3 | -31836.233333 | 2954.809605 | -1428.933333 | 1027.600000 | 0.347772 |
| 14 | 3 | -32237.566667 | 4030.489556 | -401.333333 | 2470.366667 | 0.612920 |
| 15 | 3 | -35109.266667 | 5074.418048 | -2871.700000 | — | — |

**Raw STRUCTURE output**

The raw STRUCTURE output is also available as a tab-delimited text file (for use with Excel).

| **File name** | **Run #** | **K** | **Est. Ln prob. of data** | **Mean value of Ln likelihood** | **Variance of Ln likelihood** |
| --- | --- | --- | --- | --- | --- |
| AGROAFLP_7maggio2020_run_1_f | 1 | 1 | -38556.8 | -37199.5 | 2714.5 |
| AGROAFLP_7maggio2020_run_3_f | 3 | 1 | -38586.8 | -37198.9 | 2775.8 |
| AGROAFLP_7maggio2020_run_2_f | 2 | 1 | -38583.4 | -37198.8 | 2769.2 |
| AGROAFLP_7maggio2020_run_5_f | 5 | 2 | -35038.1 | -32864.2 | 4347.8 |
| AGROAFLP_7maggio2020_run_6_f | 6 | 2 | -35064.6 | -32868.1 | 4392.9 |
| AGROAFLP_7maggio2020_run_4_f | 4 | 2 | -35157.4 | -32862.1 | 4590.5 |
| AGROAFLP_7maggio2020_run_8_f | 8 | 3 | -34318.9 | -31374.8 | 5888.3 |
| AGROAFLP_7maggio2020_run_7_f | 7 | 3 | -34932.1 | -31383.7 | 7096.7 |
| AGROAFLP_7maggio2020_run_9_f | 9 | 3 | -34178.2 | -31400.5 | 5555.3 |
| AGROAFLP_7maggio2020_run_10_f | 10 | 4 | -33518.7 | -30114.6 | 6808.1 |
| AGROAFLP_7maggio2020_run_12_f | 12 | 4 | -32918.1 | -30250.0 | 5336.1 |
| AGROAFLP_7maggio2020_run_11_f | 11 | 4 | -33329.6 | -30101.8 | 6455.5 |
| AGROAFLP_7maggio2020_run_14_f | 14 | 5 | -31871.0 | -29171.6 | 5398.7 |
| AGROAFLP_7maggio2020_run_15_f | 15 | 5 | -31872.0 | -29170.9 | 5402.1 |
| AGROAFLP_7maggio2020_run_13_f | 13 | 5 | -43766.9 | -29275.9 | 28981.9 |
| AGROAFLP_7maggio2020_run_16_f | 16 | 6 | -31293.4 | -28590.9 | 5404.9 |
| AGROAFLP_7maggio2020_run_17_f | 17 | 6 | -31140.1 | -28579.5 | 5121.3 |
| AGROAFLP_7maggio2020_run_18_f | 18 | 6 | -31264.4 | -28580.3 | 5368.1 |
| AGROAFLP_7maggio2020_run_21_f | 21 | 7 | -30814.0 | -28226.4 | 5175.3 |
| AGROAFLP_7maggio2020_run_20_f | 20 | 7 | -30878.0 | -28240.3 | 5275.5 |
| AGROAFLP_7maggio2020_run_19_f | 19 | 7 | -32026.9 | -28276.6 | 7500.7 |
| AGROAFLP_7maggio2020_run_24_f | 24 | 8 | -30705.8 | -28047.6 | 5316.5 |
| AGROAFLP_7maggio2020_run_23_f | 23 | 8 | -30641.6 | -27905.4 | 5472.5 |
| AGROAFLP_7maggio2020_run_22_f | 22 | 8 | -30668.1 | -27957.7 | 5420.9 |
| AGROAFLP_7maggio2020_run_25_f | 25 | 9 | -30941.6 | -27688.2 | 6506.6 |
| AGROAFLP_7maggio2020_run_26_f | 26 | 9 | -30221.7 | -27546.7 | 5349.9 |
| AGROAFLP_7maggio2020_run_27_f | 27 | 9 | -30209.2 | -27656.6 | 5105.3 |
| AGROAFLP_7maggio2020_run_30_f | 30 | 10 | -30119.9 | -27412.0 | 5415.8 |
| AGROAFLP_7maggio2020_run_29_f | 29 | 10 | -31094.1 | -27391.8 | 7404.4 |
| AGROAFLP_7maggio2020_run_28_f | 28 | 10 | -31261.0 | -27523.9 | 7474.2 |
| AGROAFLP_7maggio2020_run_33_f | 33 | 11 | -33381.5 | -27123.0 | 12516.9 |
| AGROAFLP_7maggio2020_run_31_f | 31 | 11 | -37796.8 | -27319.3 | 20955.1 |
| AGROAFLP_7maggio2020_run_32_f | 32 | 11 | -30345.5 | -27325.5 | 6040.0 |
| AGROAFLP_7maggio2020_run_35_f | 35 | 12 | -30257.8 | -27173.8 | 6168.0 |
| AGROAFLP_7maggio2020_run_36_f | 36 | 12 | -30793.2 | -27166.2 | 7254.0 |
| AGROAFLP_7maggio2020_run_34_f | 34 | 12 | -30170.9 | -27274.3 | 5793.3 |
| AGROAFLP_7maggio2020_run_39_f | 39 | 13 | -30654.6 | -26586.5 | 8136.3 |
| AGROAFLP_7maggio2020_run_37_f | 37 | 13 | -35199.0 | -26889.3 | 16619.3 |
| AGROAFLP_7maggio2020_run_38_f | 38 | 13 | -29655.1 | -26736.4 | 5837.4 |
| AGROAFLP_7maggio2020_run_41_f | 41 | 14 | -29783.0 | -26623.0 | 6320.0 |
| AGROAFLP_7maggio2020_run_42_f | 42 | 14 | -36889.2 | -26413.2 | 20952.1 |
| AGROAFLP_7maggio2020_run_40_f | 40 | 14 | -30040.5 | -26242.5 | 7596.1 |
| AGROAFLP_7maggio2020_run_45_f | 45 | 15 | -40393.9 | -26621.5 | 27544.7 |
| AGROAFLP_7maggio2020_run_44_f | 44 | 15 | -30275.1 | -26725.8 | 7098.5 |
| AGROAFLP_7maggio2020_run_43_f | 43 | 15 | -34658.8 | -26523.3 | 16271.2 |

**CITATION**

Earl, Dent A. and vonHoldt, Bridgett M. (2012)

STRUCTURE HARVESTER: a website and program for visualizing

STRUCTURE output and implementing the Evanno method.

Conservation Genetics Resources vol. 4 (2) pp. 359-361 doi: 10.1007/s12686-011-9548-7

Core version: vA.2 July 2014

Plot version: vA.1 November 2012

Web version: v0.6.94 July 2014

© Dent Earl 2007-2014
